# COVID-19 testing capabilities at urgent care centers in states with greatest disease burden

**DOI:** 10.12688/f1000research.23203.2

**Published:** 2020-11-16

**Authors:** Walter Hsiang, Howard Forman, Siddharth Jain, Akshay Khunte, Grace Jin, Laurie Yousman, Michael Najem, Alison Mosier-Mills, Daniel Wiznia

**Affiliations:** 1 Yale School of Medicine, New Haven, CT, 06511, USA; 2 Yale School of Management, New Haven, CT, 06511, USA; 3 Department of Radiology, Yale School of Medicine, New Haven, CT, 06511, USA; 4 Yale School of Public Health, New Haven, CT, 06511, USA; 5 Department of Orthopaedics, Yale School of Medicine, New Haven, CT, 06511, USA

**Keywords:** COVID-19, urgent care center, testing, health services

## Abstract

While rapid and accessible diagnosis is paramount to monitoring and reducing the spread of disease, COVID-19 testing capabilities across the U.S. remain constrained. For many individuals, urgent care centers (UCCs) may offer the most accessible avenue to be tested. Through a phone survey, we describe the COVID-19 testing capabilities at UCCs and provide a snapshot highlighting the limited COVID-19 testing capabilities at UCCs in states with the greatest disease burden.

## Introduction

While rapid and accessible COVID-19 diagnosis is paramount to monitoring and reducing the spread of disease, COVID-19 testing capabilities across the U.S. remain constrained. For many individuals, urgent care centers (UCCs) may offer the most accessible avenue to be tested. Using a phone survey, we describe the COVID-19 testing capabilities of UCCs in states with the greatest disease burden.

## Methods

Our study received non-human research IRB exemption from the Yale School of Medicine and participant consent was not required. We identified ten states with the highest COVID-19 caseload as of March 19, 2020 according to the Centers for Disease Control (CDC) ^1^. Using the Urgent Care Association “Find an Urgent Care” directory, we identified all UCCs within the state of interest and assigned each UCC a numeric identifier. A random number generator was used to select for a convenience sample of 25 UCCs per state. If the UCC was not able to be contacted, a new UCC was randomly selected and called. UCCs were classified into independent, hospital/health network, and academic categories.

**Figure 1.**
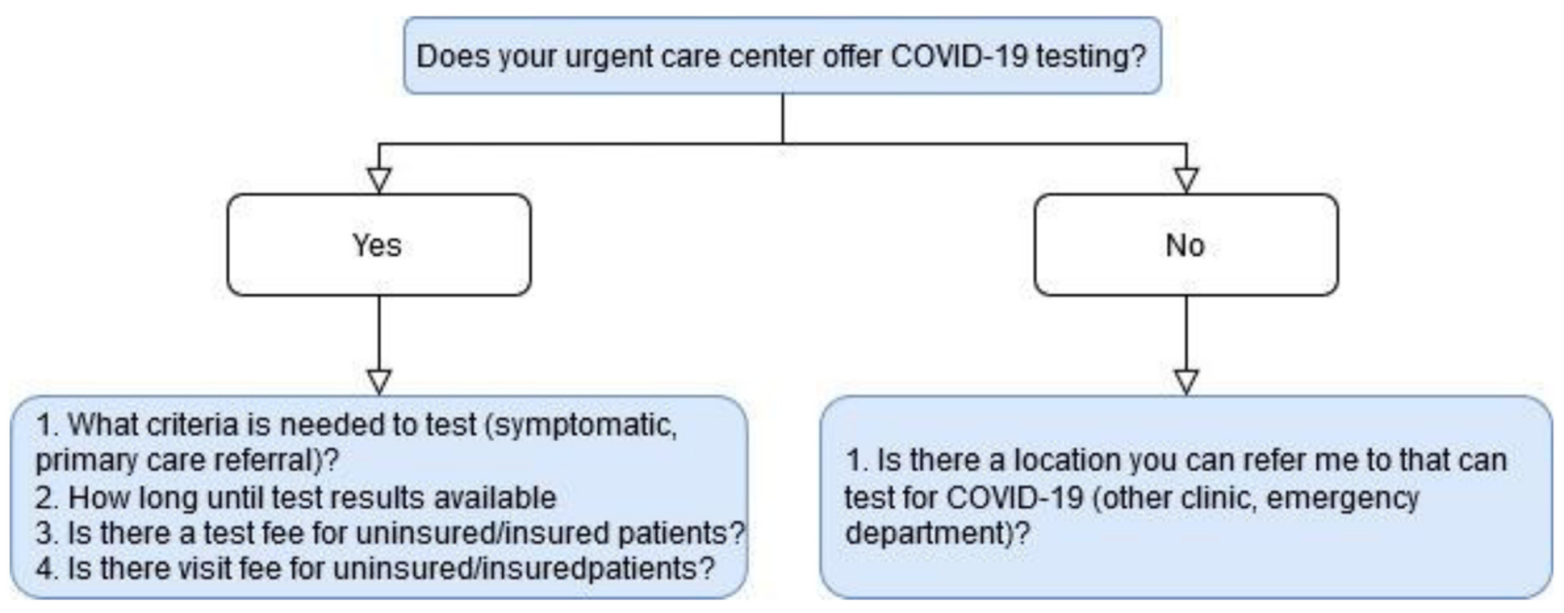
Urgent care center survey script.

Using a standardized survey script ( Figure 1), trained investigators asked UCC receptionists about COVID-19 testing ability, testing criteria, time to test results, costs of tests and visits for insured/uninsured patients, and test referrals. All 250 calls were made on March 20, 2020 and were limited to 1 minute to minimize occupying clinic resources. Using publicly available data from the United States Department of Agriculture’s Economic Research Service (based off the 2010 U.S. Census), we determined urban/rural designation based on UCC zip codes.

## Results

Of 250 UCCs contacted, 57 (22.8%) offered COVID-19 testing. Hospital/health network-affiliated UCCs were more likely to offer COVID-19 tests compared to independent UCCs (odds ratio 3.69, 95% confidence interval 1.94–7.01, p<0.0001).

Of UCCs that offered testing, 56 (98.2%) required the patient to be symptomatic (typically fever and respiratory symptoms) and 2 (0.4%) required a primary care physician referral. In total, 45 (86.5%) UCCs charged a fee to test uninsured patients, but no UCC could provide a definitive answer regarding test fees for insured patients given the shifting federal legislation. A total of 53 (94.6%) UCCs charged a visit fee in addition to the COVID-19 lab test fee. For the 49 centers that provided the wait time for test results, the median time was 120 hours (interquartile range 96 hours to 144 hours).

Of UCCs that did not offer testing, 97 (51.3%) referred individuals to other clinics that could possibly test for COVID-19, and 37 (24.8%) directly referred individuals to a specific emergency department. Individual-level results for each UCC are available as *Underlying data* ^2^.

All UCCs were located in urban-designated zip codes. 246 (98.4%) UCCs were located in metropolitan areas, while 4 (1.6%) UCCs were located in small towns with an urban cluster. 

## Discussion

In the 10 states with the greatest COVID-19 caseload, only 23% of UCCs offered COVID-19 testing. Additionally, results would take approximately five days to be processed. Although time to test results at public/state labs are typically 24–48 hours ( Table 1), time to test results at UCCs were longer as most samples are sent to external labs. However, it remains unclear whether UCC ability to obtain test samples may be unmatched by the ability to process tests. This finding underscores the importance of point-of-care testing that can rapidly detect COVID-19, particularly because severe disease peaks at approximately ten days from onset of initial symptoms ^3^.

**Table 1.** COVID-19 testing capabilities by state.

	UCCs offering tests, n (%)	Average time to test results at UCC, hours	*Average time to test results at* *state or public health lab, hours* ^*^
**California**	1 (4%)	N/A	48–72
**Colorado**	2 (8%)	108	72
**Florida**	7 (28%)	96	24–48
**Georgia**	2 (8%)	120	N/A
**Illinois**	6 (24%)	118	24
**Louisiana**	4 (16%)	138	N/A
**Massachusetts**	9 (36%)	139.2	24
**New Jersey**	7 (28%)	124.5	24–48
**New York**	10 (40%)	115.2	3–5
**Washington**	9 (36%)	91	24–48

*Time to results at state/public health labs obtained from the respective state’s Department of Public Health website as of March 20.

Fees and cost-sharing for COVID-19 tests remain unclear. The Families First Coronavirus Response Act, which passed on March 18, mandated all group and individual health plans cover COVID-19 testing and gave states the option to use Medicaid coverage for testing uninsured patients ^4^. Although this study could not definitively define test fees, most UCCs stated they would charge test fees, contrary to recent federal regulations, in addition to fees for the urgent care visit itself as of March 20. Test and visit fees at UCCs may discourage patients from seeking COVID-19 testing.

UCCs continue to face several obstacles in their ability to offer COVID-19 testing. Point-of-care rapid testing remains limited, and the necessity to externally process tests delays the receipt of test results. Overburdened healthcare providers and lack of personal protective equipment could also affect availability and costs of testing at UCCs. Our results identify several primary areas of improvement for UCCs offering COVID-19 testing: 1) the adoption of rapid point-of-care testing should be implemented and 2) UCCs should follow legislation that patients should not be charged for COVID-testing.

This report has limitations. The small number of UCCs contacted per state may not accurately represent the state’s urgent care climate. Additionally, the rapidly changing nature of the COVID-19 pandemic may affect these findings. However, this study serves as an important snapshot that highlights the limited COVID-19 testing capabilities at UCCs in the most heavily burdened states.

## Data availability

### Underlying data

Harvard Dataverse: COVID-19 Testing Capabilities at Urgent Care Centers in States with Greatest Disease Burden. https://doi.org/10.7910/DVN/SJSNZ6 ^2^.

This project contains the individual-level responses of each urgent care center to each question from the call script (JMP and XLSX file formats.)

Data are available under the terms of the Creative Commons Zero "No rights reserved" data waiver (CC0 1.0 Public domain dedication).
