# Supplementary material for: COVID-19 testing capabilities at urgent care centers in states with greatest disease burden
Source: F1000Res. 2020 Nov 16;9:328. Originally published 2020 May 4. [Version 2] doi: 10.12688/f1000research.23203.2 (PMC7689602; doi:10.12688/f1000research.23203.2)
Supplement: Peer review history

# Peer review: Reviewer response for version 2

**DOI:** 10.5256/f1000research.30654.r74893

**Published:** 2020-11-25

**Authors:** Buqing Liang

**Affiliations:** 1 Department of Neurosurgery, Baylor Scott & White Health, Temple, TX, UK

No further comments.

Is the work clearly and accurately presented and does it cite the current literature?

Yes

If applicable, is the statistical analysis and its interpretation appropriate?

Yes

Are all the source data underlying the results available to ensure full reproducibility?

Yes

Is the study design appropriate and is the work technically sound?

Partly

Are the conclusions drawn adequately supported by the results?

Partly

Are sufficient details of methods and analysis provided to allow replication by others?

Yes

Reviewer Expertise:

Neurosurgery

I confirm that I have read this submission and believe that I have an appropriate level of expertise to confirm that it is of an acceptable scientific standard.

# Peer review: Reviewer response for version 1

**DOI:** 10.5256/f1000research.25614.r68237

**Published:** 2020-07-30

**Authors:** Buqing Liang

**Affiliations:** 1 Department of Neurosurgery, Baylor Scott & White Health, Temple, TX, UK

In this study, the authors conducted a phone interview to investigate the COVID-19 testing capabilities at urgent care centers in states with the greatest disease burden. This is an interesting and meaningful topic, especially during this pandemic crisis. However, there are some issues that need to be addressed.

- How did the authors validate the data from the UCCs to ensure no wrong information was conveyed? Even at the same center, two receptionists may give different answers to the same question. Is it possible to double confirm with two different receptionists?
- As the authors state that UCCs were randomly selected. How to differentiate big city centers vs rural area centers? High burden states still have rural areas that may have a very low incidence of COVID infection that does not supplement with UCCs with test capability. What is the percentage of these UCCs?
- Based on the above-mentioned reason, wouldn't investigating UCCs in cities with high COVID-19 incidence be a more reasonable design?
- Lastly, in the discussion part, the authors merely analyzed the results but did not put forward how it can be utilized for improvement. Namely, what's the meaning of this study?

Is the work clearly and accurately presented and does it cite the current literature?

Yes

If applicable, is the statistical analysis and its interpretation appropriate?

Yes

Are all the source data underlying the results available to ensure full reproducibility?

Yes

Is the study design appropriate and is the work technically sound?

Partly

Are the conclusions drawn adequately supported by the results?

Partly

Are sufficient details of methods and analysis provided to allow replication by others?

Yes

Reviewer Expertise:

Neurosurgery

I confirm that I have read this submission and believe that I have an appropriate level of expertise to confirm that it is of an acceptable scientific standard, however I have significant reservations, as outlined above.

# Response

**Published:** 2020-11-05

**Authors:** Walter Hsiang

**Affiliations:** Yale University School of Medicine, USA

How did the authors validate the data from the UCCs to ensure no wrong information was conveyed? Even at the same center, two receptionists may give different answers to the same question. Is it possible to double confirm with two different receptionists?

**We cannot guarantee that receptionists may or may not convey the correct information, but contacting offices in a randomized approach with a large sample has been a validated way to collect survey information. We cannot double confirm with receptionists given the changing landscape since March. However, this caller approach has been used and validated in many our similar survey methodologies: doi.org/10.1097/SLA.0000000000004373; doi.org/10.1177/0046958019838118; doi.org/10.1016/j.surg.2018.03.013; doi.org/10.1016/j.arth.2015.03.015**

 As the authors state that UCCs were randomly selected. How to differentiate big city centers vs rural area centers? High burden states still have rural areas that may have a very low incidence of COVID infection that does not supplement with UCCs with test capability. What is the percentage of these UCCs?

**We have added an urban vs rural variable in our analysis based off the UCC’s zip code in the 2010 U.S. Census. UCCs were all located in urban-designated areas. The urban-designation can be broken down into subcategories, and 4 centers were located in smaller towns with an urban cluster. However, these UCCs are still considered urban. It is well known that UCCs tend to locate in wealthier, urban areas.**

**The results section and data sharing section have been updated accordingly.**

 Based on the above-mentioned reason, wouldn't investigating UCCs in cities with high COVID-19 incidence be a more reasonable design?

**Given that the primary locations of UCCs at the time of the study were in urban metropolitan areas, we feel confident that our sample essentially represented cities with high COVID-19 incidence.**

 Lastly, in the discussion part, the authors merely analyzed the results but did not put forward how it can be utilized for improvement. Namely, what's the meaning of this study?

**Thank you for this feedback. We have added two areas of improvement in the 3 ^rd^ paragraph of the discussion.**

# Peer review: Reviewer response for version 1

**DOI:** 10.5256/f1000research.25614.r67613

**Published:** 2020-07-21

**Authors:** Siddharth Sridhar

**Affiliations:** 1 Department of Microbiology, University of Hong Kong, Pokfulam, Hong Kong

This study provides a snapshot of the COVID-19 testing landscape in ambulatory clinics in the United States in March 2020. Overall, only 22.8% of UCCs offered COVID-19 testing. Turn around time was up to several days in several centres. 

 Testing and tracing are vital means to control community COVID-19 epidemics. Testing is the first step in the test-isolate-quarantine paradigm that has successfully contained COVID-19 in many parts of the world. Deployment of testing to a wide section of symptomatic individuals in the communities is a challenge. UCCs are a potentially convenient location where such testing can be performed; widespread testing at these centres would also generate vital surveillance data. The fact that less than a quarter of all UCCs in affected states offered COVID-19 testing represents a lost opportunity and a lesson for future pandemic preparedness.  

 Some suggestions for improvement are as follows: 

1. This data is now 4 months old. While it would be too much to ask to repeat the survey, it would be good if the authors can provide any available updates on the number of UCCs offering COVID-19 testing or turn around time of state laboratories. Is the low availability of testing at UCCs improving in these states, especially in heavily affected areas like Florida? 
2. Briefly discuss the obstacles to UCCs offering COVID-19 tests. 
3. How does this snapshot in March reflect the situation today in July? Do the authors observe an association between a higher proportion of testing UCCs in March with better COVID-19 control today? 

Is the work clearly and accurately presented and does it cite the current literature?

Yes

If applicable, is the statistical analysis and its interpretation appropriate?

Yes

Are all the source data underlying the results available to ensure full reproducibility?

Yes

Is the study design appropriate and is the work technically sound?

Yes

Are the conclusions drawn adequately supported by the results?

Yes

Are sufficient details of methods and analysis provided to allow replication by others?

Yes

Reviewer Expertise:

Emerging infectious diseases; clinical virology; hepatitis E

I confirm that I have read this submission and believe that I have an appropriate level of expertise to confirm that it is of an acceptable scientific standard.

# Response

**Published:** 2020-11-05

**Authors:** Walter Hsiang

**Affiliations:** Yale University School of Medicine, USA

This data is now 4 months old. While it would be too much to ask to repeat the survey, it would be good if the authors can provide any available updates on the number of UCCs offering COVID-19 testing or turn around time of state laboratories. Is the low availability of testing at UCCs improving in these states, especially in heavily affected areas like Florida? 

**While it is difficult to repeat this study under the previous condition, we attempted to provide additional clarity regarding this question by repeating the phone calls to the same 25 UCC locations in Florida in October. In Florida, the availability of testing improved from 28% to 64%, however, the mean wait time increased from 4 days to 7 days.**

 Briefly discuss the obstacles to UCCs offering COVID-19 tests. 

**We have added several statements on the obstacles to UCC testing in 3 ^rd^ paragraph of the discussion section.**

 How does this snapshot in March reflect the situation today in July? Do the authors observe an association between a higher proportion of testing UCCs in March with better COVID-19 control today? 

**It is difficult to say whether higher availability of testing is associated with better COVID-19 control, since numerous factors affect the ability to control the spread of infection. However, as we continue into an additional wave of COVID-19 infections, sufficient availability of and accessibility to testing remains paramount.**
